# Regular Meeting, May 24, 1875

**Published:** 1875-06-01

**Authors:** E. Warren Sawyer


### REGULAR MEETING, MAY 24, 1875.

*Reported by E. Warren Sawyer, M.D.*

PROF. THOMAS BEVAN, President, in the chair.

Prof. Freer reported a series of experiments made upon dogs, with a view to ascertaining how long blood may be kept, and afterwards safely transfused into dogs.

*First Experiment.*—Ten ounces of venous, defibrinated, dog's blood was transfused into another's veins, the blood having stood for seven hours upon ice, but warmed to 95° F. just before the operation. No bad symptoms resulted.

*Second Experiment.*—Eight ounces of arterial blood, which had been exposed for seventeen hours to the temperature of the laboratory, about 65° F., was transfused with no ill effect.

*Third Experiment.*—This dog was first bled from the carotid artery, till he could bleed no longer; about twenty ounces being let. Fresh blood was transfused, and dog recovered rapidly.

*Fourth Experiment.*—First bled to insensibility; respiration had ceased; thirty-two ounces escaping; ten ounces of blood which had previously stood upon ice for forty-eight hours, was transfused. Dog was resuscitated, but died seventeen hours afterward. This was an unfavorable subject for the operation, as it was a dog which had been caught up from the street at the moment, and was greatly frightened.

*Fifth Experiment.*—This dog was first bled six ounces; then seven ounces of blood, which had been on ice for the previous seventy-two hours, was transfused. Dog recovered.

In all instances, the blood used was defibrinated immediately after being drawn. With one exception, the blood was brought to 95° F. at the moment of transfusion. In this exceptional instance, the blood was accidentally passed into the dog's veins cold, without any bad effect.

Dr. Freer has long been of the opinion, that blood was not as highly vitalized as other tissues; he regarded it rather as food in its most highly prepared shape, and ready for assimilation. By transfusion, the body can be nourished *direct*, independently of the digestive organs. The indication for this operation will be the threatened inanition of the patient. More people die from inanition than we realize. In pulmonary consumption, for example, the patient rarely dies from asphyxia, and very rarely from hæmorrhage, but from the failure of the digestive organs to furnish assimilable food; in other words, from starvation.

In caries about large joints, the patient ultimately succumbs, because the digestive function has failed long before. By supplying the readily assimilated food in the operation of transfusion, we give the digestive organs rest; and in some instances, we may hope for an arrest in the morbid changes, even in so formidable a disease as pulmonary consumption.

In answer to Dr. Starkweather, McDonald's apparatus was used in transfusion.

Answer to Prof. Etheridge. No unsuccessful cases thus far have occurred.

Dr. Simons remarked, that in cases of immediate danger of starvation, small quantities of this blood food might be given, from time to time, just as one would inject hydrate of chloral in the veins, or any other drug.

The President regarded the thought as a most important one; for, in some instances of malignant disease of the digestive tract, which seems alone too slight to cause death, then the threatened starvation might be averted almost indefinitely.

In answer to Dr. Hamill, Dr. Freer said, that if a person should digest no food at all, it will not be necessary to resort to transfusion daily, for the good effects are not so transitory.

Dr. Simons suggested, that it would be an interesting fact to determine by experiment, viz., how long a dog could be kept alive by continuous transfusions, with absolutely no food taken into the stomach.

It was moved by Dr. Hyde, and carried, that Prof. Freer be requested to read a more extended paper on the subject, and exhibit apparatus, details of the operation, etc., at some future day.

Dr. Lester Curtis read a paper on the histological structure of the serous membranes, with reference to certain pathological processes which they may be the seat of. The subject was illustrated with diagrams. The writer had been struck by the fact, that the effect of inflammation of the serous membrane was quite unlike the effect of that process when the mucous membrane was attacked. An explanation of this disparity was to be found in the structure of these membranes. The epithelial covering of the serous membrane can be separated into two layers, between which, coagulable lymph was effused, thus converting the membrane into a true granulating surface, which is always followed by contraction. Unlike this, the epithelial covering of the mucous membrane is not thus separable into two layers.

Dr. Henrotin reported the case of a lady who had suffered a scald of the feet, about two weeks before. Everything was progressing favorably, the healing nearly completed, when she was seized, while sitting in her chair, with palpitation of the heart, and syncope, followed by death in twenty minutes. During life, the lady had frequent attacks of palpitation of the heart, and syncope; once a blowing sound was heard over the aortic orifice.

Dr. Hyde reported a case of pityriasis versicalor in a lady, which he felt emboldened to speak of, because two well-known physicians of this city had treated her with various drugs in large quantities, for liver disease, and finally, for kidney trouble. A single warm bath, and the local application of sulphite of soda in solution, was all that was required.

Dr. Wood mentioned, that he had seen thirty cases of this disease in recruits. He had not found it intractable under the use of corrosive sublimate externally.

Dr. Simons spoke of a case in which the hand was passed into the rectum for the purpose of diagnosis. The case was obscure; that of a man who passed masses of fæces through the urethra.

